# Genome-Wide Association Studies and Comparison of Models and Cross-Validation Strategies for Genomic Prediction of Quality Traits in Advanced Winter Wheat Breeding Lines

**DOI:** 10.3389/fpls.2018.00069

**Published:** 2018-02-02

**Authors:** Peter S. Kristensen, Ahmed Jahoor, Jeppe R. Andersen, Fabio Cericola, Jihad Orabi, Luc L. Janss, Just Jensen

**Affiliations:** ^1^Nordic Seed A/S, Odder, Denmark; ^2^Department of Molecular Biology and Genetics, Center for Quantitative Genetics and Genomics, Aarhus University, Tjele, Denmark; ^3^Department of Plant Breeding, The Swedish University of Agricultural Sciences, Alnarp, Sweden

**Keywords:** wheat quality, Zeleny sedimentation, thousand-kernel weight, falling number, genomic selection, GBLUP, Bayesian Power Lasso

## Abstract

The aim of the this study was to identify SNP markers associated with five important wheat quality traits (grain protein content, Zeleny sedimentation, test weight, thousand-kernel weight, and falling number), and to investigate the predictive abilities of GBLUP and Bayesian Power Lasso models for genomic prediction of these traits. In total, 635 winter wheat lines from two breeding cycles in the Danish plant breeding company Nordic Seed A/S were phenotyped for the quality traits and genotyped for 10,802 SNPs. GWAS were performed using single marker regression and Bayesian Power Lasso models. SNPs with large effects on Zeleny sedimentation were found on chromosome 1B, 1D, and 5D. However, GWAS failed to identify single SNPs with significant effects on the other traits, indicating that these traits were controlled by many QTL with small effects. The predictive abilities of the models for genomic prediction were studied using different cross-validation strategies. Leave-One-Out cross-validations resulted in correlations between observed phenotypes corrected for fixed effects and genomic estimated breeding values of 0.50 for grain protein content, 0.66 for thousand-kernel weight, 0.70 for falling number, 0.71 for test weight, and 0.79 for Zeleny sedimentation. Alternative cross-validations showed that the genetic relationship between lines in training and validation sets had a bigger impact on predictive abilities than the number of lines included in the training set. Using Bayesian Power Lasso instead of GBLUP models, gave similar or slightly higher predictive abilities. Genomic prediction based on all SNPs was more effective than prediction based on few associated SNPs.

## Introduction

Wheat (*Triticum aestivum* L.) is a major cereal crop that is grown in most parts of the world. In 2014, more than 700 million tons of wheat was produced globally (FAOSTAT, 2016). Depending on quality, wheat can be used as animal feed or for human consumption in a variety of products, such as bread, biscuits, and noodles (Shewry, 2009). Wheat quality, therefore, is determined by many traits, e.g., grain protein content, gluten composition, and grain hardness. The special viscoelastic properties of wheat dough are mainly due to the gluten content, which consist of a network of high- and low-molecular weight (HMW and LMW) glutenins and monomeric gliadins. The majority of the grain protein in wheat is gluten protein (approximately 80%), and a high grain protein content is associated with high wheat quality. However, the composition of glutenin subunits and gliadins is also important for the wheat quality (Shewry 2009). Gluten content and quality can be estimated using the Zeleny sedimentation test. Here, flour is mixed with lactic acid, causing the gluten to expand and sediment. Large sedimentation-volumes indicate high gluten content and strength (Peña, 2002). The major glutenin loci are the HMW glutenins *Glu-A1, Glu-B1*, and *Glu-D1* located on the long arm of chromosome 1A, 1B, and 1D, respectively, whereas the LMW glutenins *Glu-A3, Glu-B3*, and *Glu-D3* are located on the short arm of 1A, 1B, and 1D, respectively. The *Glu-3* loci each consist of several alleles encoding LMW glutenin subunits, and they are linked to the gliadin loci *Gli-A1, Gli-B1*, and *Gli-D1* (Liu et al., 2014). Genetic variation in these complex loci contributes to wheat quality, but other factors than gluten content also affect the quality (Liu et al., 2014). Grain hardness is an essential trait for the milling properties and end-use of wheat. Grain with hard endosperm texture are preferred for making bread, as flour made from these have a higher water absorption capacity as a result of increased starch granule damage during the milling. The lower water absorption of flour from soft grain is favorable for the production of cookies and cakes. The puroindoline genes, *Pina-D1* and *Pinb-D1*, at the *Hardness* locus on chromosome 5DS account for most of the genetic variation for grain hardness. However, grain hardness is also affected by the *GSP-1* (*Grain Softness Protein*) gene at the same locus and by minor QTL (Quantitative Trait Loci) on other chromosomes. Deletions or knock-out mutations in either of the *Pin* genes lead to increased hardness (Bhave and Morris, 2008; Pasha et al., 2010).

Test weight (the weight of 100 L of grain) is a grain quality trait that is important to many end-users (Bordes et al., 2014). The test weight and the grain yield component thousand-kernel weight (TKW) are often used as indicators of flour yield, although these two traits are not always strongly correlated with flour yield (Hook, 1984). Another factor that influences wheat quality is falling number. Falling number is an indicator of the activity of the starch-degrading enzyme α-amylase, and it is measured as the time it takes a viscometer stirrer to fall through a heated, gelatinized suspension of flour and water. Low falling number (high α-amylase activity) is associated with pre-harvest sprouting of the grain, which has a significant negative impact on quality. Flour from grain with low falling number generally gives soft, sticky dough and smaller bread loaves (Mares and Mrva, 2008).

Phenotyping of quality traits is expensive and time-consuming. Consequently, most wheat breeders prioritize the yield of new lines over improvement of grain quality. Furthermore, it is difficult to phenotype most quality traits in the early stages of a breeding program, because relatively large quantities of grain are needed for the tests. Thus, reliable genetic markers for quality traits are useful as indicators of the grain quality of wheat lines. DNA can be extracted from few grain or leaf samples, and genotyping of genetic markers are in many cases economically advantageous compared to phenotyping (Heffner et al., 2011).

A number of genes/QTL affecting quality traits in wheat have been found across the genome (for reviews see e.g., Liu et al., 2014; Varzakas et al., 2014). However, many of these QTL are population or environment specific and can therefore not easily be applied in other breeding programs. In traditional marker-assisted selection, only few markers linked to QTL with large effects are used. However, for many quantitative traits, the majority of the QTL have small effects, so marker-assisted selection might not be very effective (Bernardo, 2008). For genomic selection, lines are genotyped for thousands of DNA markers across the genome in order to capture many QTL with small effects as well (Meuwissen et al., 2001). This can improve the predictive ability for quantitative traits that are controlled by many genes. One way to implement genomic selection is to use a training set with lines, which have both been genotyped and phenotyped, to develop a model for predicting GEBVs (Genomic Estimated Breeding Values) of lines in a validation set, where only the genotypes are known. The genomic information can also be used to increase the accuracy of estimated breeding values of phenotyped individuals that are genetically related. Genomic selection have so far mainly been implemented in animal breeding programs, especially for cattle breeding (Hayes et al., 2009), but much research is being devoted to genomic selection in plant breeding (Heslot et al., 2015), including wheat and barley (Battenfield et al., 2016; Michel et al., 2016; Nielsen et al., 2016; Cericola et al., 2017). However, the best strategy of implementing genomic selection in wheat breeding programs is not clear (Bassi et al., 2015). The training set has a major impact on the predictive abilities, but the optimal size and composition of the training set might vary between traits or populations. Similarly, the type of model used for genomic predictions can be optimized for different cases.

SNP-BLUP (Best Linear Unbiased Prediction) models are commonly used for genomic selection. In these multiple regression models, a high number of SNP effects are fitted simultaneously (Meuwissen et al., 2001). GEBVs are calculated as the sum of additive SNP effects, which are usually assumed to be from a Normal distribution. The SNP effects are shrunken equally toward zero to fit with the total genetic variance and to avoid over-fit in the models due to including a very high number of markers. GBLUP (Genomic BLUP) models are equivalent to SNP-BLUP models, but GEBVs are calculated using a variance-covariance matrix with genetic relationship between lines based on the SNPs (Meuwissen et al., 2001; VanRaden, 2008). Since all SNP effects are shrunken equally in GBLUP, large SNP effects might be shrunken too much, while small effects are not shrunken enough. Thus, this might not be the best way to estimate SNP effects for traits, where most SNPs have no effect and some SNPs have large effects. To improve the modeling of the genetic architecture, Bayesian models can be used. Here, SNP effects can easily be assigned other types of distributions than the Normal distribution. When using more heavy-tailed distributions than the Normal distribution, large effects are shrunken less and small effects more. However, Bayesian models require longer computation times than BLUP models, and often do not perform considerably better, if the genetic relationship between lines is high (Meuwissen et al., 2001; VanRaden, 2008).

The objectives of the present study were to identify genetic loci associated with the quality traits grain protein content, Zeleny sedimentation value, test weight, falling number, and TKW, and to develop models for genomic prediction of these quality traits using GBLUP and Bayesian Power Lasso models. Genomic predictions based on all SNPs were compared with predictions based on few SNPs with most significant effects.

## Materials and methods

### Plant material

A total of 635 F_6_ winter wheat lines from two breeding cycles (set2014 and set2015) of the Danish plant breeding company Nordic Seed A/S were used for the analyses. The lines of the first breeding cycle, set2014, consisted of 321 lines and were harvested in 2014, and the lines of the second breeding cycle, set2015, consisted of 314 lines that were harvested in 2015. In total, 96 different lines were used as crossing parents in the two sets, and 6 of these lines were used as parents in both sets. The number of different full-sib families from the crosses was 159, and the number of lines in each full-sib family ranged from 1 to 33 with an average of 10 lines (Table 1). Each line was grown in a single plot of 9.9 m^2^ at Lolland in Denmark following standard Danish agricultural practices. During the growth season, approximately 180 kg of nitrogen were applied per hectare, and no irrigation was used.

**Table 1:** Distribution of lines in full-sib families.

Full-sibs per family	1	2	3	4	5	6	7	8	9	10	11	12	13	15	18	33
Number of families	39	27	21	29	9	10	5	5	3	2	3	2	1	1	1	1
Total number of lines	39	54	63	116	45	60	35	40	27	20	33	24	13	15	18	33

### Phenotyping

The wheat lines were phenotyped for grain protein content, Zeleny sedimentation value, test weight, falling number, and TKW (Supplementary Material). Grain protein content was determined using Near Infrared Transmission (Infratec™ 1241 Grain Analyser with Test Weight Module, FOSS, Denmark), so that test weight was also measured. TKW was determined by image analysis using the Seed Analyzer MARVIN (GAT Sensorik GmbH, Germany) and SeedCount SC5000 (Next Instruments, Australia). Grain was milled using a Quadrumat Junior mill (Brabender GmbH & Co. KG, Germany) to obtain flour for Zeleny sedimentation, and using a Lab Mill 3100 (Perten, Sweden) for falling number. Zeleny sedimentation tests for indication of gluten content and gluten strength were done using the international standard method ISO 5529, and falling number was measured using a Falling Number 1900 System (Perten, Sweden), method ISO/DIS 3093.

### Genotyping

DNA was extracted from leaves of three bulked, 2-week old seedlings for each line using a modified CTAB method (Rogers and Bendich, 1985). The lines were genotyped by TraitGenetics (Germany) using the 15K Illumina Infinium iSelect HD Custom Genotyping BeadChip technology. A total number of 13,006 SNP markers were called, and 10,802 of these were selected for the analyses after editing for minor allele frequency (MAF) lower than 1% and for more than 10% missing values (Supplementary Material). For all lines, more than 90% of the SNPs were successfully genotyped. The number of SNPs mapped to each chromosome is shown in Table 2.

**Table 2:** Number of SNPs mapped to each of the 21 chromosomes.

Chromosome	1A	2A	3A	4A	5A	6A	7A	Total, A
No. of SNPs	474	474	489	319	559	557	657	3529
Chromosome	1B	2B	3B	4B	5B	6B	7B	Total, B
No. of SNPs	736	713	692	355	797	685	565	4543
Chromosome	1D	2D	3D	4D	5D	6D	7D	Total, D
No. of SNPs	269	217	123	49	133	128	114	1033

*Total number of SNPs: 10,802. Unmapped: 1697*.

### Statistical analyses

Genome-wide association analyses were conducted by single marker regression using the following model that was run for each of the 10,802 SNPs:

(1) $$y=Xb+w_{i}a_{i}+Z_{1}u+e$$

where ***y*** is a vector of observed phenotypes, ***X*** and ***Z***_1_ are design matrices, ***b*** is a vector of fixed effects (mean and year/set), ***w***_*i*_ is the vector of genotypes of the *ith* SNP coded as 1, 0, −1, *a*_*i*_ is the additive genetic effect of the *ith* SNP, ***u*** is a vector of additive genetic effects of the lines [***u*** ~ *N(0*,***G***$\sigma_{g}^{2}$*)*, where ***G*** is a G-matrix and $\sigma_{g}^{2}$ is additive genetic variance], and ***e*** is a vector of random residual effects (***e*** ~ *N(0*,***I***σ_*e*_^2^), where **I** is an identity matrix and σ_*e*_^2^ is the residual variance). The effects of year and set (breeding cycle) could not be separated, since each line was only tested 1 year. Software package DMU was used for estimation of model effects and variance components by restricted maximum likelihood (Madsen and Jensen, 2013).

G-matrices with genomic relationship between the lines were used to correct for family structure in order to avoid spurious associations (Price et al., 2010). For each chromosome, a G-matrix was calculated based only on the markers that were mapped to the remaining chromosomes. This G-matrix was then used for the structure-correction of the SNPs mapped to the excluded chromosome, in order to ensure that effect of the SNP was not included in the model twice. The G-matrices were calculated using method one proposed by VanRaden (2008):

(2) $$G=\frac{Z_{2}Z_{2}^{\prime }}{2\sum p_{i}(1-p_{i})}$$

where *p*_*i*_ is MAF of *ith* marker, ***Z***_2_ = ***M*** – ***P***, ***M*** is a matrix with the alleles of the markers coded as 1, 0, −1 (missing genotypes were set to 0), and ***P*** is a matrix with MAF calculated as 2(*p*_*i*_ – 0.5).

Population structure was studied by performing a principal component analysis on the G-matrix computed from all 10,802 SNPs using the built-in R function “prcomp” (R Development Core Team, 2016). A heat map and dendrogram were constructed based on the G-matrix using the R function “heatmap” (R Development Core Team, 2016).

Average genetic distances were calculated from the 10,802 SNPs using the R function “dist” (R Development Core Team, 2016) and dividing the obtained Euclidean distances with $\sqrt{2*10,802}$ to get the modified Rogers' distance (Reif et al., 2005).

Genomic inflation factors, λ_IF_, were calculated for each trait and used to correct the *p*-values for inflation (Hinrichs et al., 2009). The inflation factor λ_IF_ was calculated by dividing the observed median value of the chi-squared statistic for the SNPs with the expected median value. The expected value is based on the assumption that there are no associations between the SNPs and the trait. Inflated *p*-values can be caused by e.g., population structure, which gives λ_IF_ values of more than 1. If λ_IF_ were above 1, the chi-squared statistics were divided by λ_IF_ and then used to calculate the *p*-values. To reduce the risk of false-positive SNP-trait associations, a Bonferroni correction was used to set the significance threshold at 5% divided by number of SNPs (0.05/10,802 = 4.6^*^10^−6^).

GWAS were also performed by fitting all SNPs at the same time with a Bayesian Power Lasso model using the Bayz software (Janss, 2011):

(3) $$y=Xb+Z_{3}u+e$$

where ***y*** is a vector of observed phenotypes, ***b*** is a vector of the mean + year/set effect with design matrix **X**, ***Z***_3_ is a matrix of the alleles of the SNPs coded as 0, 1, 2, ***u*** is a vector of additive genetic SNP effects, and ***e*** is a vector of residual effects. Residuals were assigned a Normal prior distribution. The residual variance, the mean, year/set effect, and rate parameter, λ_*RP*_, were assigned flat prior distributions. The prior distribution of SNP effects was assigned to be an exponential power distribution:

(4) $$p\left(u\right)=\prod_{i=1}^{m}\frac{1}{2}\lambda_{RP}e^{-\lambda_{RP}{\left|u_{i}\right|}^{\beta }}$$

where *m* is number of markers and β is shape parameter to control the sparsity, which affects the shrinkage of the SNP effects. When β is set to 1, the model is equivalent to the standard Bayesian Lasso, where the absolute SNP effects, |*u*_*i*_|, are assumed to follow an exponential distribution. In this case, large effects are shrunken a bit less and small effects are shrunken a bit more than in the Normal distribution. Setting β to less than 1, increases the difference between markers with large and small effects even further, as it is |*u*_*i*_|^β^ that follow an exponential distribution (Gao et al., 2013). In the analyses performed here, models were tested with β set to 0.2, 0.4, 0.8, and 1.0. Deviance Information Criterion was calculated for each of the models and used to choose the optimal β for each of the traits (Spiegelhalter et al., 2002). Model parameters were estimated using Markov Chain Monte Carlo (MCMC) with a length of 100,000 of which 30,000 cycles were the burn-in. Posterior means were computed using the tool pbayz supplied with Bayz, and convergence was checked using the R package CODA (Plummer et al., 2006).

Genomic predictions using all SNPs were performed using the Bayesian Power Lasso model (3) and using a GBLUP model:

(5) $$y=Xb+Z_{4}u+e$$

where ***y*** is a vector of observed phenotypes, ***X*** and ***Z***_4_ are design matrices, ***b*** is a vector of fixed effect (mean and year/set), ***u*** is a vector of additive genetic effects (***u*** ~ *N(0*,***G***$\sigma_{g}^{2}$*)*, where ***G*** is a G-matrix computed as above (2) using all 10,802 SNPs and $\sigma_{g}^{2}$ is additive genetic variance), and ***e*** is a vector of random residual effects (***e*** ~ *N(0*,***I***σ_*e*_^2^)).

Estimation of model effects and variance components for the GBLUP and Bayesian Power Lasso models were done using DMU and Bayz, respectively. For the GBLUP models, the narrow sense genomic heritability (de los Campos et al., 2013) based on records of single plots was calculated as:

(6) $$h^{2}=\frac{d(G)\sigma_{g}^{2}}{d(G)\sigma_{g}^{2}+\sigma_{e}^{2}}$$

where *d(**G**)* is the average diagonal element of the G-matrix (calculated using all SNPs), $\sigma_{g}^{2}$ is additive genetic variance and $\sigma_{e}^{2}$ is residual variance.

For the Bayesian Power Lasso models, *h*^2^ was calculated by dividing the additive genetic variance (variance of GEBVs) with the phenotypic variance.

Predictive abilities of the models were determined as the correlations between observed phenotypes corrected for fixed effects and GEBVs. These correlations were compared with the square root of the narrow sense genomic heritability, which is the maximum correlation that can be achieved. Bias in the genomic predictions was calculated as the slope of the regression line of the corrected phenotypes on the GEBVs. The expectation of this slope is 1.0, and the bias is the deviation from this expectation.

Several types of cross-validation strategies were tested to study the effectiveness of different approaches for implementation of genomic selection in breeding programs:

- LOO (Leave-One-Out), where the GEBV of each line was predicted based on the rest of the lines. The LOO strategy was used to study the predictive ability when using the largest training set available and the highest possible genetic relationship between lines in the training and validation set.
- LFO (Leave-Family-Out), where the GEBVs of lines in each half-sib family were predicted based on lines from other half-sib families, so that they had no parents in common. The LFO cross-validation strategy was used to study the effect of the genetic relationship between the lines in training and validation sets.
- LSO (Leave-Set-Out), where the GEBVs of lines in each set were predicted based on lines only from the other set. The LSO cross-validation strategy was used to study the predictive ability when predicting GEBVs of lines from one breeding cycle based on lines from another breeding cycle.
- k-fold, where the lines were randomly divided into k folds (2, 5, or 10) of equal size and the GEBVs of lines in each fold were predicted based on lines only in the other folds. The size of the training sets in the 2-, 5-, and 10-fold cross-validations were approximately 318 lines, 508 lines, and 572 lines, respectively. The k-fold cross-validation strategy was used to study the effect of the size of the training set.

Predictions of breeding values were also performed using the three and the ten best SNPs for each trait according to the single marker GWAS or the Bayesian Power Lasso (model 1 or 3). Here, the 635 lines were randomly divided into 5-folds of equal size. The best SNPs and their effects were estimated based on the lines in four of the folds and used for predictions of breeding values in the fifth fold. This was done to avoid unrealistically high predictive abilities that would be the result of using the same lines for estimation and prediction. The best SNPs were defined as the SNPs most significantly associated with the studied trait for the single marker regressions, and as the SNPs with the largest additive genetic effects for the Bayesian Power Lasso. When selecting SNPs using model 1, maximum one SNP was selected from each chromosome to avoid selecting several SNPs linked to the same QTL. Predictions were made using the SNP effects estimated in model 1. SNP effects were also re-estimated as random effects by fitting the selected SNPs simultaneously in either model 3 or in a SNP-BLUP model (equivalent to GBLUP model 5). Predictions were evaluated by calculating the correlation between observed phenotypes corrected for fixed effects and estimated breeding values.

## Results

### Phenotyping

**Figure 1:**
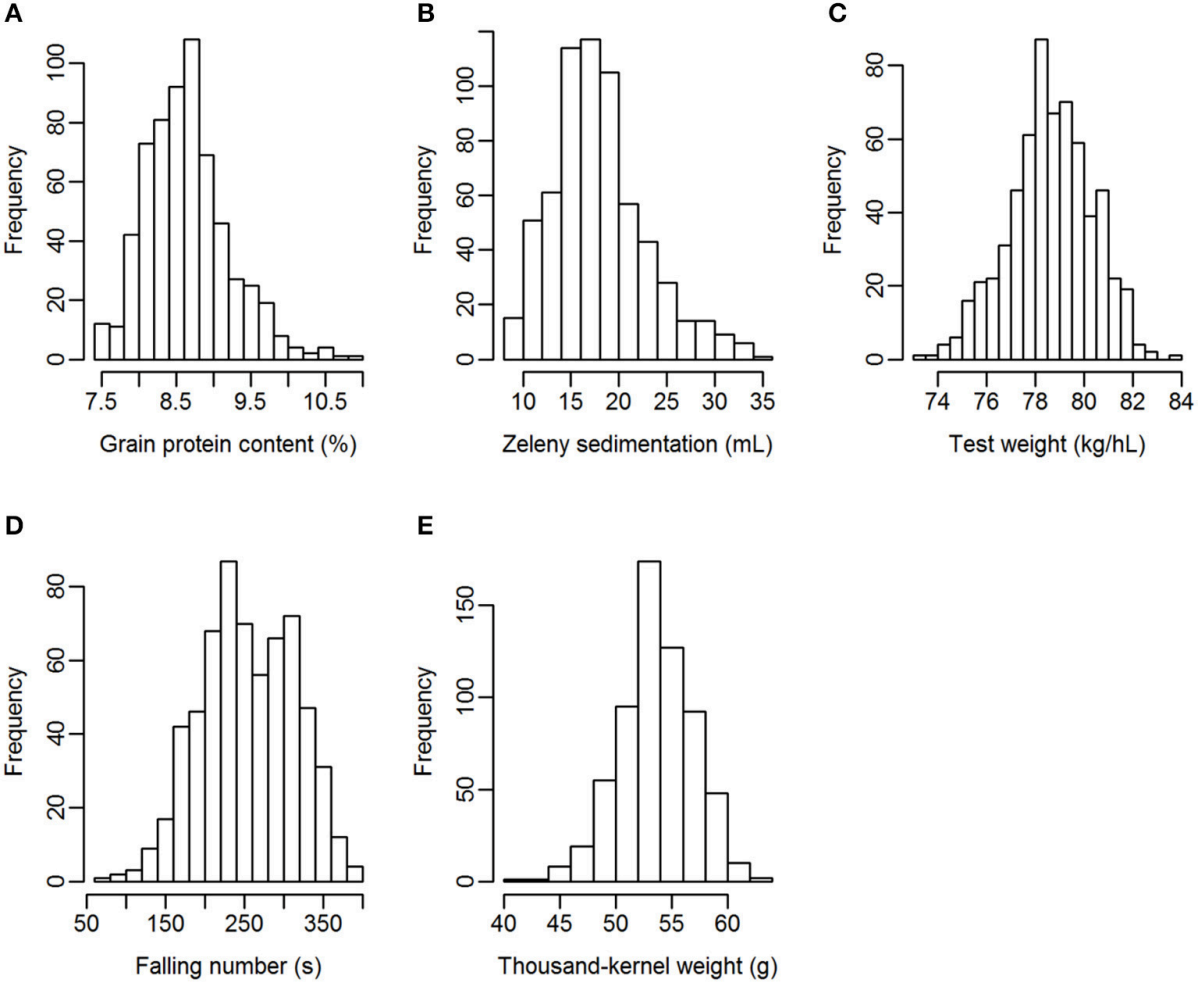
Distribution of the phenotypes. **(A)** Grain protein content, **(B)** Zeleny sedimentation, **(C)** test weight, **(D)** falling number, **(E)** thousand-kernel weight.

Grain from 635 winter wheat lines were phenotyped for the quality traits grain protein content, Zeleny sedimentation value, test weight, falling number, and TKW. The lines were from two different breeding cycles (set2014 and set2015). Phenotypic variation was observed for all traits, especially for Zeleny sedimentation and falling number, which had a coefficient of variation of 26.9 and 23.4%, respectively (Table 3). The phenotypes appeared to be approximately normally distributed for all traits (Figure 1).

**Table 3:** Mean, range, and coefficient of variation (CV) for phenotypic data.

**Phenotype**	**Mean**	**Range**	**CV (%)**
Grain protein content (%)	8.7	7.5–10.9	6.4
Zeleny sedimentation (mL)	18.3	8.0–36.0	26.9
Test weight (kg/hL)	78.7	73.4–83.7	2.2
Falling number (s)	254.7	79.0–391.0	23.4
Thousand-kernel weight (g)	53.7	40.8–63.0	6.0

### Genotyping

**Figure 2:**
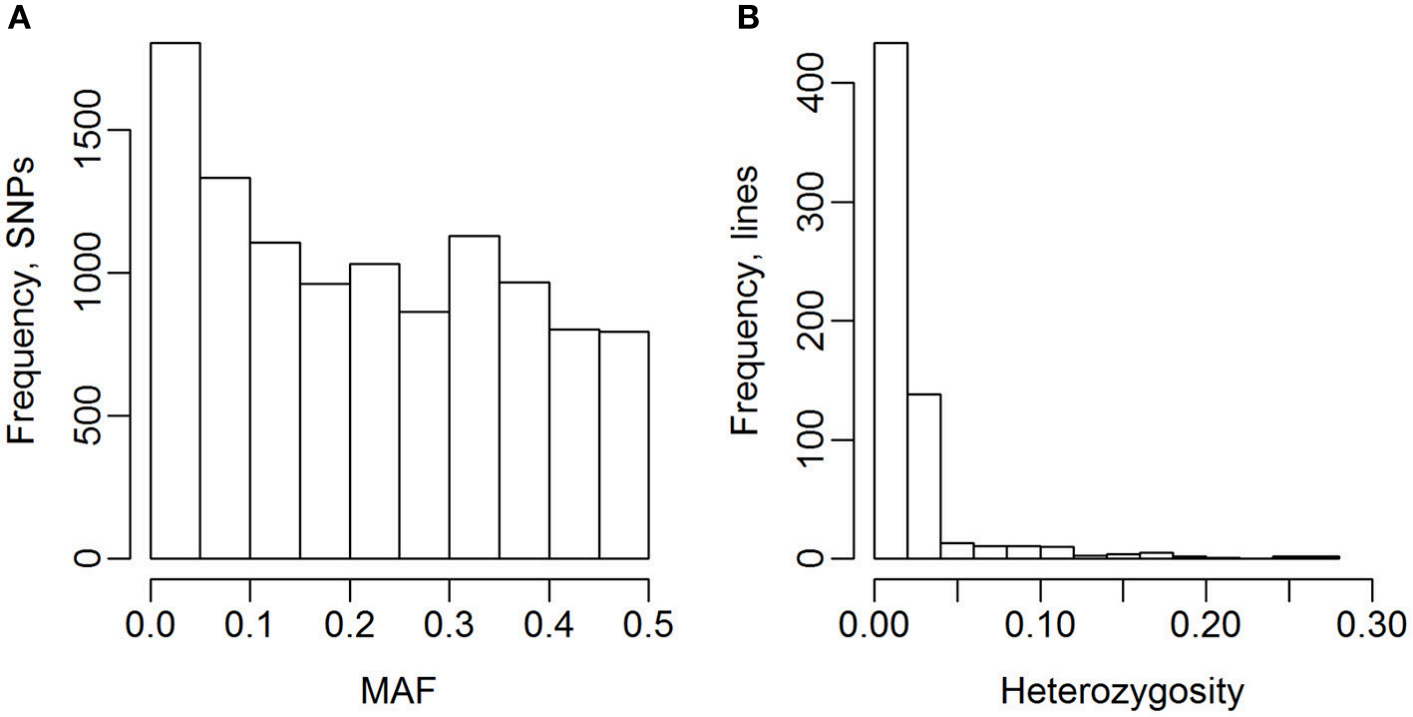
Minor allele frequency (MAF) of SNPs and heterozygosity of lines. **(A)** MAF after selection of SNPs. **(B)** Heterozygosity of the 635 wheat lines.

The 635 lines were genotyped for 13,006 SNP markers, and 10,802 of these were selected for the analyses (Table 2). The genotype for 141 of the 13,006 SNPs were missing for more than 10% of the lines, and 2,063 SNPs had a MAF lower than 1%, so they were excluded from the analyses. The MAF distribution of the selected SNPs are shown in Figure 2A. The average degree of heterozygosity of the lines was 2.5%, and the majority of the lines were heterozygous for less than 5% of the SNPs (Figure 2B).

**Figure 3:**
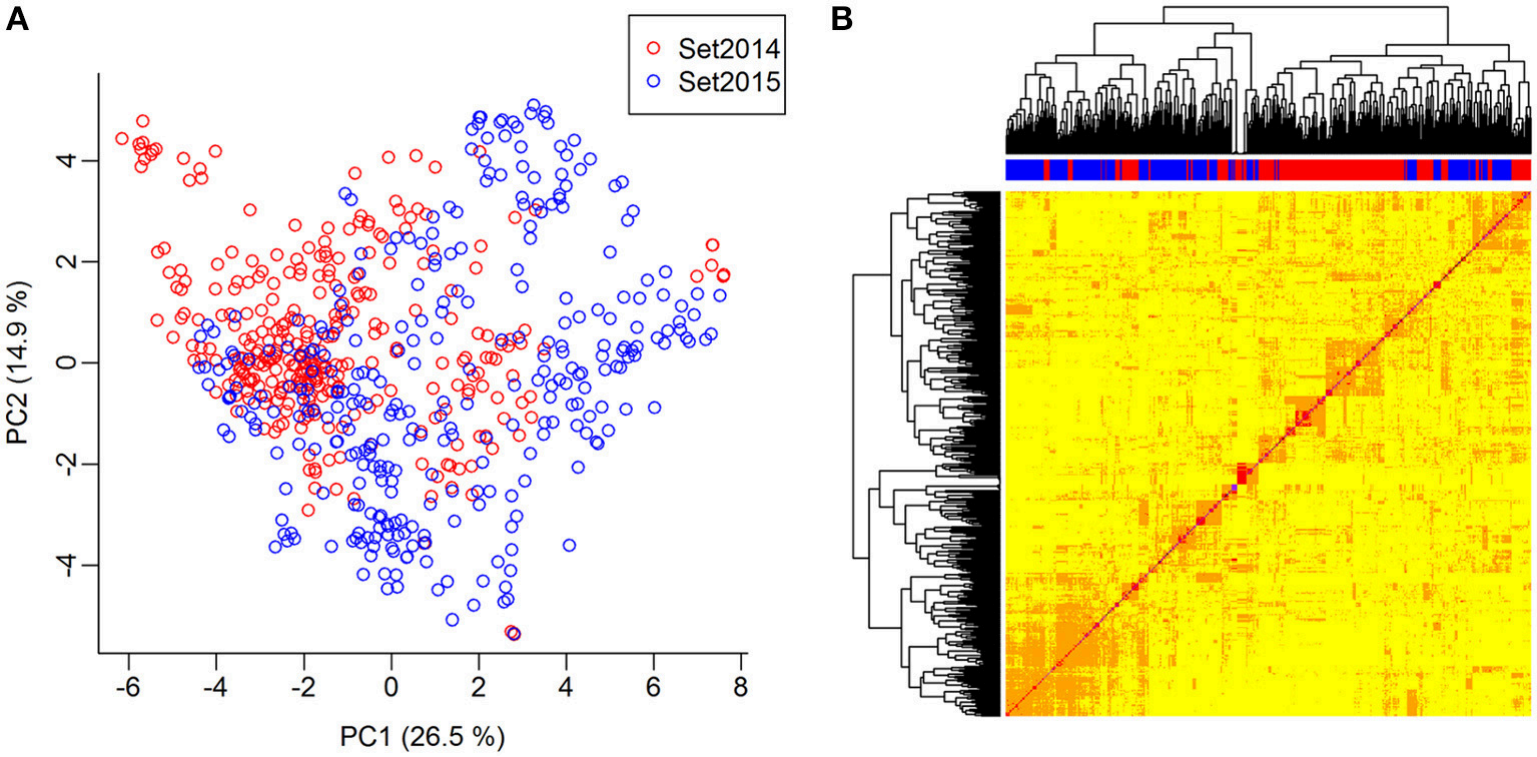
Genomic relationship between lines. **(A)** Principal component analysis of the G-matrix for the 635 lines. Plot of principal components 1 and 2, which explain 26.9 and 14.6% of the variance, respectively. Lines from set2014 are displayed in red, while lines from set2015 are displayed in blue. **(B)** Heat map and dendrogram of G-matrix showing the relationship between the 635 lines based on the genotyped SNP markers. The bar on top of the heat map shows, which set the lines, are from: Red: Lines from set2014, blue: Lines from set2015.

The genomic relationship between the lines was determined by computing a G-matrix based on the 10,802 SNPs. The lines were genetically related both within and between the two sets. The lines of set2014 were not clearly separated from the lines of set2015 in the principal component analysis of the G-matrix (Figure 3A). Furthermore, several groups of lines with close genetic relationships were revealed in the heat map of the G-matrix, but no lines were clearly genetically different from the rest of the lines (Figure 3B). The average genetic distance calculated as modified Rogers' distance was 0.74 both within set2014 and within set2015. The average genetic distance between set2014 and set2015 was 0.76.

Narrow sense genomic heritabilities (based on single plots) and variance components based on the GBLUP and Bayesian Power Lasso models are shown in Table 4. Heritabilities ranged from 0.56 for protein to 0.81 for test weight and TKW based on the GBLUP models.

**Table 4:** Variance components, their standard errors, and narrow sense genomic heritabilities estimated from the GBLUP and from the Bayesian Power Lasso models.

**Phenotype**	**GBLUP**			**Bayesian Power Lasso**		
	**Additive genetic variance**	**Residual variance**	**h^2^**	**Additive genetic variance**	**Residual variance**	**h^2^**
Grain protein content	0.16 ± 0.015	0.12 ± 0.011	0.56	0.13 ± 0.015	0.13 ± 0.013	0.51
Zeleny sedimentation	15.4 ± 1.01	4.2 ± 0.49	0.78	20.3 ± 0.85	4.1 ± 0.46	0.83
Test weight	2.2 ± 0.14	0.51 ± 0.064	0.81	2.0 ± 0.11	0.51 ± 0.070	0.79
Falling number	2, 555 ± 184	825 ± 95	0.75	2, 575 ± 151	815 ± 107	0.76
Thousand-kernel weight	10.3 ± 0.69	2.4 ± 0.31	0.81	7.4 ± 0.49	2.3 ± 0.36	0.76

### GWAS: single marker regression

**Figure 4:**
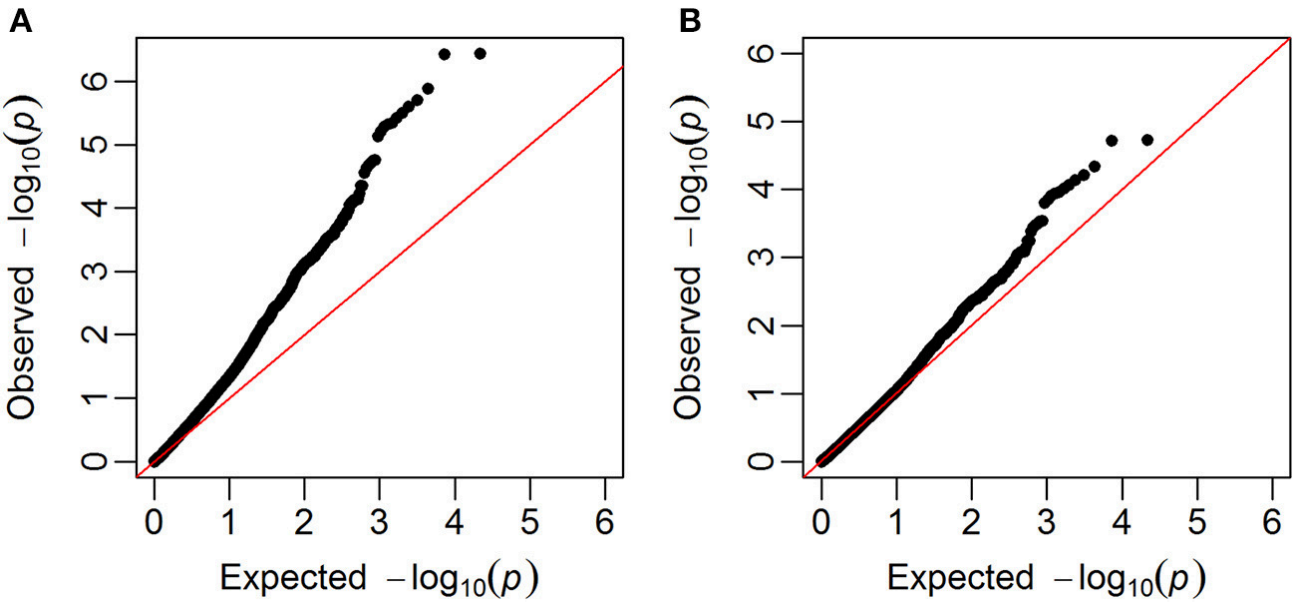
Q-Q plots. Plots of observed –log_10_(*p*-values) against expected given no associations between SNPs and the trait (in this example falling number). **(A)** Before the correction with the genomic inflation factor, **(B)** after the correction with the genomic inflation factor.

GWAS were carried out using single marker regression of each marker on the phenotypes. Family structure was taken into account using G-matrices based on all chromosomes, except the one for the selected marker. Q-Q plots of observed against expected –log_10_(*p*-values) showed that the observed *p*-values were inflated, i.e., most of the *p*-values were lower than the *p*-values expected for SNPs that were not associated with the studied trait. The genomic inflation factor (λ_IF_) (Hinrichs et al., 2009), which ranged from 1.29 to 1.78, were therefore used to correct the *p*-values, so that they were closer to the expectation (example in Figure 4).

**Figure 5:**
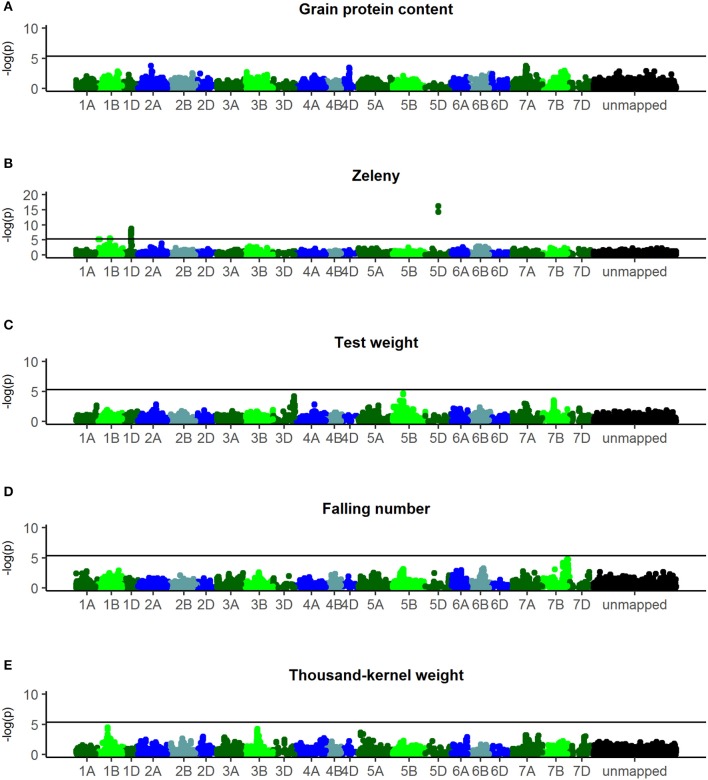
Manhattanplots of –log_10_(*p*-values). **(A)** Grain protein content, **(B)** Zeleny, **(C)** test weight, **(D)** falling number, **(E)** thousand-kernel weight. Last bin is unmapped SNPs.

Manhattan plots of the corrected –log_10_(*p*) are shown in Figure 5. For Zeleny sedimentation, three regions with significant SNPs were found on chromosomes 1B (*p*-value = 2.5^*^10^−6^), 1D (*p*-value = 1.6^*^10^−9^), and 5D (*p*-value = 6.1^*^10^−17^) (Figure 5B). Based on the most significant SNPs, these regions explained 3.2, 6.5, and 9.2% of the genetic variance for Zeleny sedimentation, respectively. The frequencies of the alleles associated with higher Zeleny sedimentation were 64% (1B), 14% (1D), and 28% (5D).

For falling number, a nearly significant region was found on chromosome 7B (*p*-value = 4.3^*^10^−5^) with an allele frequency of 89% for the advantageous allele (Figure 5D). No significant SNPs were found for falling number when analyzing all 635 lines, but using only lines from set2014 (321 lines) revealed one significant SNP on chromosome 4DS (*p*-value = 1.1^*^10^−6^) with an advantageous allele frequency of 77% (results not shown).

For TKW, a region on chromosome 1B was nearly significant (*p*-value = 3.4^*^10^−5^) and explained 1.7% of the genetic variance (Figure 5E). The advantageous allele frequencies of the SNPs in this region were 97%. No significant regions were identified for grain protein content and test weight (Figures 5A,C).

### GWAS: Bayesian Power Lasso

**Figure 6:**
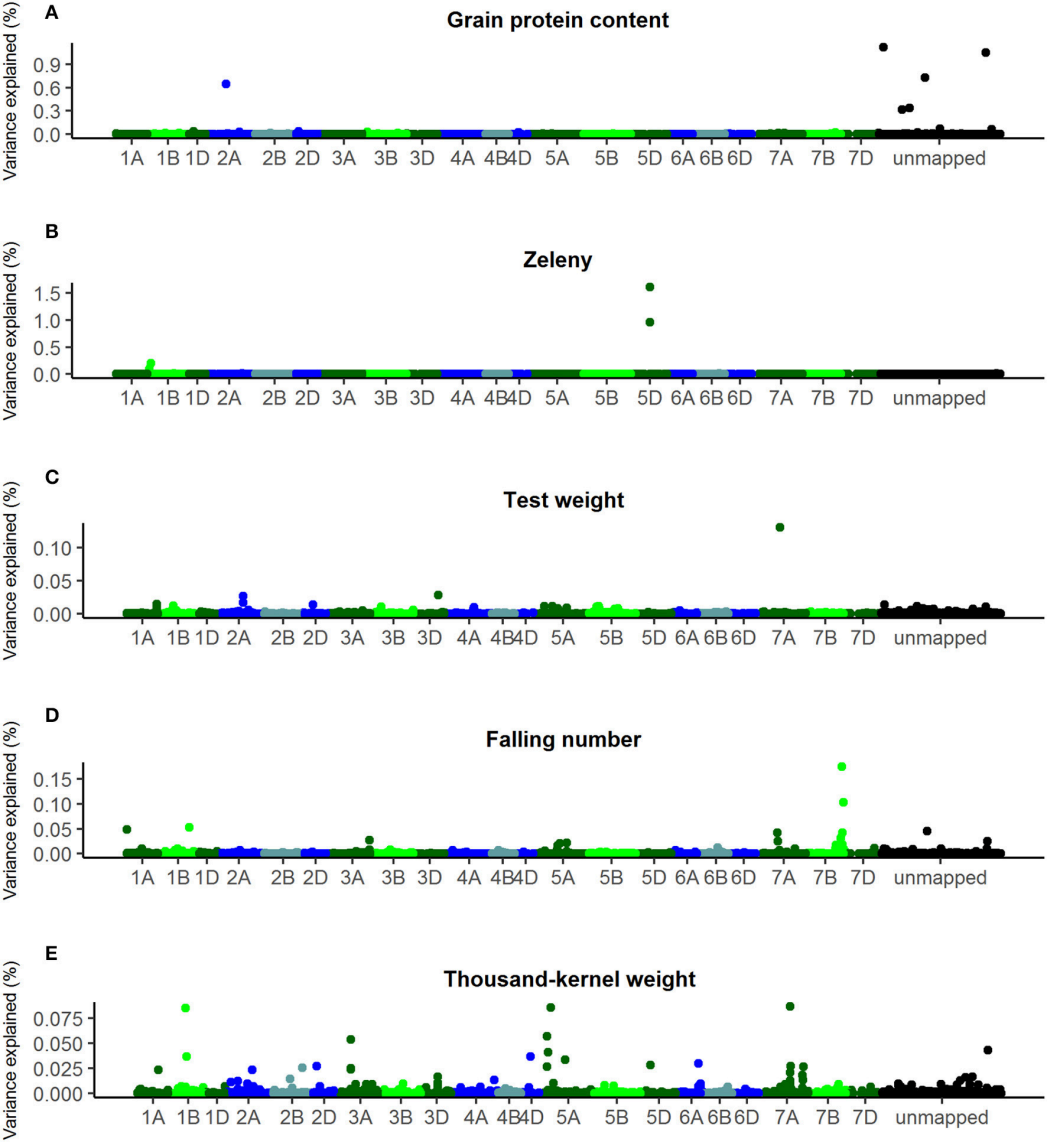
Percentage of additive genetic variance explained by the SNPs according to the Bayesian Power Lasso analyses. **(A)** Grain protein content, **(B)** Zeleny, **(C)** test weight, **(D)** falling number, **(E)** thousand-kernel weight. Last bin is unmapped SNPs.

Another approach for GWAS was also used, where all SNPs were fitted at the same time using a Bayesian Power Lasso model, where SNP effects were assumed to be from an exponential power distribution (Gao et al., 2013). The optimal value for the shape parameter, β, of the Bayesian Power Lasso models was determined by comparing the Deviance Information Criterion of models with shape parameter 0.2, 0.4, 0.6, 0.8, and 1. For each of the studied traits, the shape parameter giving the lowest Deviance Information Criterion was chosen: 0.2 for grain protein content, 0.4 for TKW and falling number, and 0.6 for Zeleny sedimentation and test weight. Plots of the additive genetic variance explained by the SNPs according to these models are shown in Figure 6.

The SNPs that explain most variance according to the Bayesian analyses were located in the same genetic regions as the most significant SNPs identified by single marker regression for Zeleny sedimentation, TKW, and falling number. However, the explained variance was lower compared with the single marker regressions. For the Bayesian analyses, only one SNP explaining more than 1.5% of the additive genetic variance was found (SNP on chromosome 5D associated with Zeleny sedimentation). Sorting the lines based on their genotypes for the associated SNPs for Zeleny sedimentation on chromosome 5D, 1D, and 1B, and looking at the mean of the phenotypic data, showed that these SNPs had a clear effect on Zeleny sedimentation (Table 5).

**Table 5:** Mean values for Zeleny sedimentation and number of lines with the different alleles of the top SNPs on chromosome 5D, 1D, and 1B.

**A**	**Genotype**	**Zeleny mean**	**No. of lines**	**B**	**SNP 5D**	**SNP 1D**	**SNP 1B**	**Zeleny mean**	**No. of lines**
SNP 5D	T	22.1	166		T	G	A	28.4	28
	C	16.8	442				G	21.7	7
SNP 1D	G	23.7	82			A	A	21.5	101
	A	17.4	538				G	16.9	21
SNP 1B	A	19.3	389		C	G	A	21.7	11
	G	16.5	215				G	19.7	11
						A	A	16.8	214
							G	15.9	164

*Green color indicates positive alleles. Zeleny sedimentation mean for all 635 lines was 18.3 mL*.

***(A)** Values based only on one of the SNPs. **(B)** Values based on the different combinations of the three SNPs*.

For test weight, the SNP with the largest effect were located on chromosome 7A, but the SNP only explained 0.13% of the genetic variance. For grain protein content, one SNP on chromosome 2A and five unmapped SNPs were identified to have the largest effects. However, together these SNPs explained less than 5 % of the total genetic variance.

### Genomic prediction

**Figure 7:**
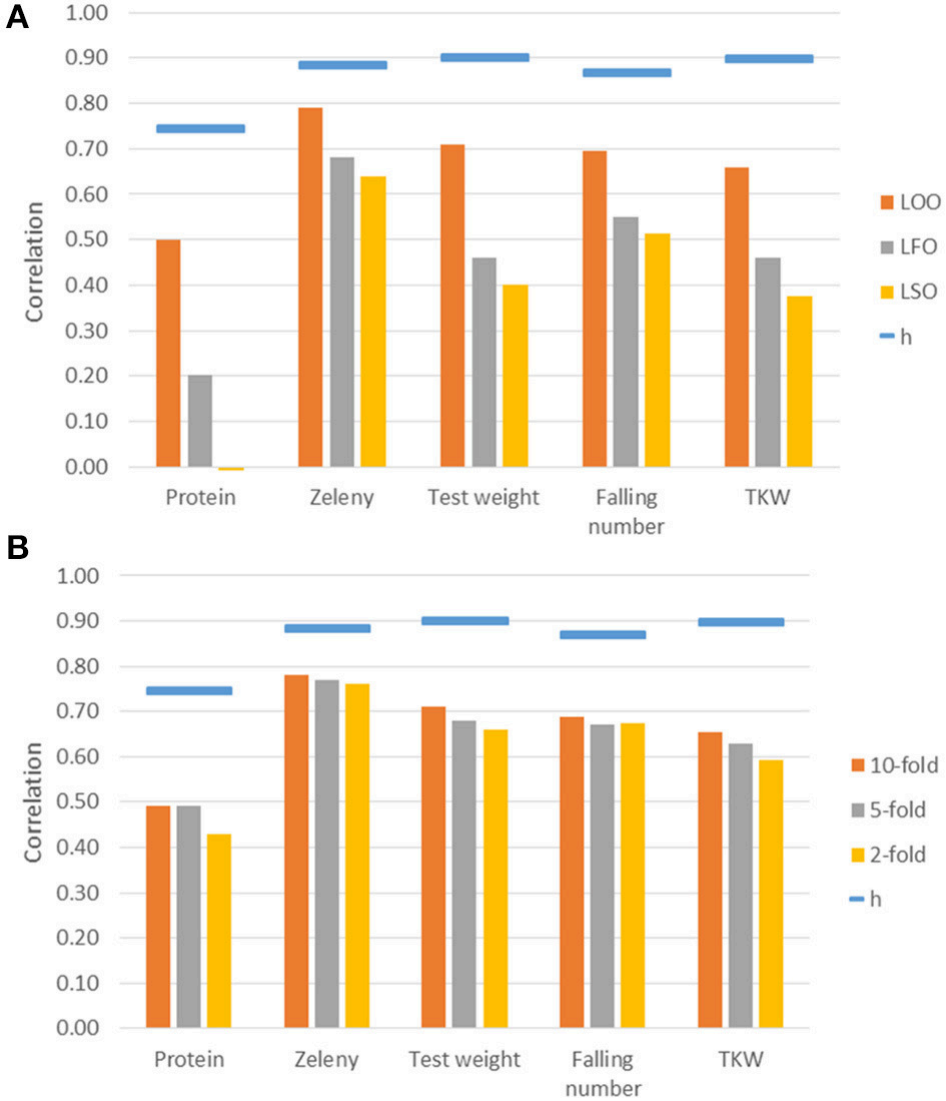
Correlations between observed phenotypes corrected for fixed effects and GEBVs based on the GBLUP models. Maximum correlation, h, is the square root of the narrow sense genomic heritability and is shown as blue bars over the correlations. Correlations are based on different kinds of cross-validations. **(A)** Leave-One-Out, Leave-Family-Out, Leave-Set-Out, and **(B)** k-fold cross-validations.

Genomic predictions were conducted based on all 10,802 SNPs, and the predictive ability was evaluated using different kinds of cross-validations. The correlations between observed phenotypes corrected for fixed effects and GEBVs using GBLUP models are shown in Figure 7. The highest correlations were for Zeleny, where the correlation for LOO (0.79) was quite close to the maximum (the square root of the narrow sense heritability). The lowest correlations were for protein content, where the prediction of GEBVs across sets did not work (LSO, correlation: −0.01). For all traits, the correlations based on the LOO cross-validation was higher than those based on the LFO and LSO (Figure 7A). The k-fold cross-validation strategy showed that smaller training sets resulted in slightly lower correlations (Figure 7B). The predictive abilities based on the LFO ranged from 0.2 for grain protein content to 0.68 for Zeleny sedimentation. The LSO cross-validations resulted in the lowest predictive abilities for all traits.

Genomic predictions were also performed using the Bayesian Power Lasso model. The predictive abilities were very close to or slightly better than the predictions based on the GBLUP model (Table 6). The largest improvement was for the LSO cross-validations for Zeleny sedimentation, where the predictive ability increased from 0.64 to 0.70, when using the Bayesian model instead of the GBLUP model.

**Table 6:** Comparison of predictive abilities based on GBLUP and Bayesian Power Lasso models.

		**h**	**LSO**	**2-fold**	**5-fold**	**10-fold**
Grain protein content	GBLUP	0.75	−0.01	0.43	0.49	0.50
	Bayesian	0.71	−0.02	0.44	0.48	0.50
Zeleny sedimentation	GBLUP	0.88	0.64	0.76	0.77	0.78
	Bayesian	0.92	0.70	0.80	0.81	0.82
Test weight	GBLUP	0.90	0.40	0.66	0.68	0.71
	Bayesian	0.89	0.40	0.66	0.69	0.71
Falling number	GBLUP	0.87	0.51	0.67	0.67	0.69
	Bayesian	0.87	0.51	0.68	0.68	0.69
Thousand-kernel weight	GBLUP	0.90	0.37	0.59	0.63	0.65
	Bayesian	0.87	0.38	0.59	0.63	0.66

*Predictive abilities are presented as correlations between phenotypes corrected for fixed effects and GEBVs. Maximum correlation, h, is the square root of the narrow sense genomic heritability. Cross-validations: LSO: Leave-Set-Out, and k-fold*.

The GEBVs predicted based on the LOO cross-validations were either not or only slightly biased (regressions from 0.93 for falling number to 0.98 for Zeleny sedimentation, Table 7). However, for the LFO and LSO, there was some bias for all traits except Zeleny sedimentation, so the scale of the predicted and observed phenotypes did not completely match. For the k-fold cross-validations, most regressions were close to 1. The most biased of the k-folds was the 2-fold cross-validation for protein with a regression of 0.89, and the remaining regressions were all higher than 0.9. The biases for the 5- and 2-folds were in most cases slightly worse than for the 10-folds.

**Table 7:** Regressions of corrected phenotypes on GEBVs and their standard errors based on different cross-validations strategies using the GBLUP models.

	**LOO**	**LFO**	**LSO**	**2-fold**	**5-fold**	**10-fold**
Grain protein content	0.96 ± 0.06	0.53 ± 0.10	−0.02 ± 0.11	0.89 ± 0.07	0.94 ± 0.06	0.97 ± 0.06
Zeleny sedimentation	0.98 ± 0.03	1.02 ± 0.04	1.05 ± 0.05	0.99 ± 0.03	0.98 ± 0.03	0.97 ± 0.03
Test weight	0.95 ± 0.04	0.71 ± 0.05	0.70 ± 0.06	0.91 ± 0.04	0.93 ± 0.04	0.95 ± 0.04
Falling number	0.93 ± 0.04	0.88 ± 0.05	0.83 ± 0.05	0.92 ± 0.04	0.91 ± 0.04	0.92 ± 0.04
Thousand-kernel weight	0.96 ± 0.04	0.73 ± 0.06	0.66 ± 0.06	1.01 ± 0.05	0.95 ± 0.05	0.97 ± 0.04

*Cross-validations: LOO, Leave-One-Out; LFO, Leave-Family-Out; LSO, Leave-Set-Out; and k-fold*.

In total, 128 of the 635 lines were selected (mainly based on yield) to continue to F_7_ in the breeding program. The selected lines were phenotyped for Zeleny sedimentation, TKW, and falling number again in the F_7_ generation. The correlation between the phenotypic F_7_ data and GEBVs estimated from the F_6_ data using LSO cross-validations was 0.68 for Zeleny sedimentation, 0.35 for TKW, and 0.41 for falling number.

### Predictions based on most significant SNPs

The three and ten best SNPs were identified for each trait using the single marker regression models and the Bayesian Power Lasso models. The correlation between observed phenotypes corrected for fixed effects and GEBVs based on these SNPs were calculated to compare the models.

**Figure 8:**
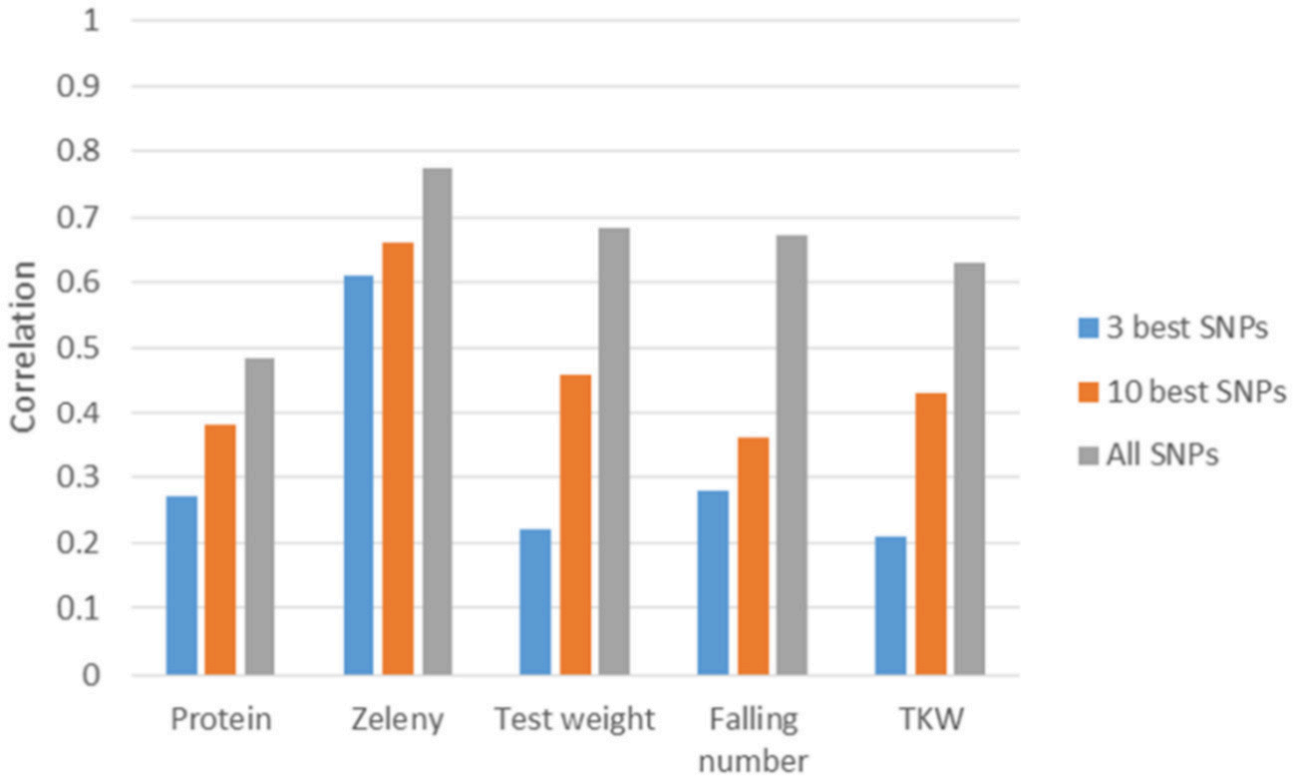
Correlations between observed phenotypes corrected for fixed effects and GEBVs based on 5-fold cross-validations. GEBVS were predicted from the three or ten best SNPs based on single marker regression (effects re-estimated with SNP-BLUP) and from all 10,802 SNPs based on the GBLUP model.

The correlations based on the best SNPs according to the single marker regression were higher than the correlations based on the Bayesian Power Lasso for grain protein content, test weight, falling number, and TKW (Table 8). For Zeleny sedimentation, the correlations were very similar for both models. In most cases, predictions improved a bit when using the SNP effects re-estimated in a SNP-BLUP model rather than using SNP effects estimated from the single marker regression or Bayesian Power Lasso model. For all traits, the predictions improved when using the ten best SNPs instead of the three best. Correlations were higher for all traits when using all 10,802 SNPs for predictions compared to using the three or ten best SNPs (Figure 8).

**Table 8:** Correlations between observed phenotypes corrected for fixed effects and breeding values predicted based on the best three or ten SNPs according to the single marker regression and to the Bayesian Power Lasso.

	**Top 3 SNPs**				**Top 10 SNPs**			
**SNP selection**	**Single marker regression**		**Bayesian Power Lasso**		**Single marker regression**		**Bayesian Power Lasso**	
**Effect re-estimation**	**Single marker regression**	**SNP-BLUP**	**Bayesian Power Lasso**	**SNP-BLUP**	**Single marker regression**	**SNP-BLUP**	**Bayesian Power Lasso**	**SNP-BLUP**
Grain protein content	0.21	0.27	0.11	0.14	0.34	0.38	0.25	0.28
Zeleny sedimentation	0.61	0.61	0.59	0.59	0.62	0.66	0.68	0.69
Test weight	0.26	0.22	0.16	0.18	0.42	0.46	0.19	0.29
Falling number	0.17	0.28	0.17	0.14	0.41	0.36	0.34	0.17
Thousand-kernel weight	0.24	0.21	0.09	0.24	0.31	0.43	0.18	0.43

## Discussion

Elite breeding material was used in the present study, so the number of significant SNPs (and their effects) were low compared with other studies, where the plant material had high diversity and larger phenotypic differences between lines (Sun et al., 2008; Bordes et al., 2014; Zanke et al., 2015). Furthermore, QTL with large effects on the traits may be fixed in the breeding lines, and will therefore not be detectable. However, there was considerable phenotypic and genetic variation for all the studied traits, indicating that several unfixed QTL were present in the material. Wheat quality traits can be significantly influenced by environmental effects and GxE interactions. Previous studies that have used multi-environment field trials showed that many QTL were only identified in certain locations, years or populations (Deng et al., 2015; Jin et al., 2016; Krystkowiak et al., 2017). Thus, QTL identified in the present study are not necessarily effective in all environments. Heritabilities for wheat quality traits reported in other studies are generally intermediate or high, but varies depending on populations, environments, and experimental setups (Mohler et al., 2014; Battenfield et al., 2016; Michel et al., 2016). Here, the heritability was lowest for grain protein content (0.56 according to GBLUP model) and highest for test weight and TKW (0.81 for both). The high heritabilities indicate that major parts of the phenotypic variation are caused by genetic variation. Thus, SNPs associated with the traits may be identified, and genomic selection could potentially be used for improvement of the traits at this stage of the breeding program.

The wheat lines have been grown with low nitrogen fertilization (to follow the Danish agricultural practice), so the grain protein content was quite low. No SNPs were significantly associated with grain protein content, suggesting that this trait is controlled by many QTL with small effects. Furthermore, the relatively low heritability indicate that protein content is strongly affected by environmental effects and GxE interactions (Shewry, 2009; Michel et al., 2016). The wheat lines would have to be grown in several locations to account for these effects. The Bonferroni corrected significance threshold might be too conservative, since many of the SNPs were in linkage disequilibrium (but were regarded as individual tests), so non-significant SNPs were not necessarily without effect on the traits. However, using false discovery rate instead only resulted in a higher number of significant SNPs for Zeleny sedimentation, and these SNPs were located in the same regions as the SNPs already identified using the Bonferroni correction (results not shown).

The SNPs most significantly associated with grain protein content were located on chromosome 2A, 4D, and 7A. In agreement with the present study, Groos et al. (2003) identified QTL associated with grain protein content on these three chromosomes, but also on chromosome 3A and 7D. QTL on chromosome 3B, 5A, and 6A were identified by Sun et al. (2008). Hence, grain protein content seems to be controlled by many QTL across the genome. Other studies indicate that this may also be the case for other quality traits. Zanke et al. (2015) found QTL associated with TKW on all chromosomes, while Bordes et al. (2014) found QTL associated with test weight on all chromosomes, exept chromosome 5D. In contrast, Krystkowiak et al. (2017) and Liu et al. (2016) each identified only one QTL significantly associated with test weight (located on chromosome 3B and 2B, respectively). Liu et al. (2016) also identified a QTL with large effect on TKW on chromosome 3B, but did not find major QTL for six other quality traits. Most significant QTL for TKW were identified on chromosome 1A, 4A, and 7A, and for protein content on chromosome 1D and 5D by Krystkowiak et al. (2017).

Few SNPs were mapped to the D-genome chromosomes compared with the chromosomes of the A- and B-genome both in Bordes et al. (2014) and in the present study (Table 2). The hybridization event(s) that introduced the D-genome into wheat is thought to have happened a long time after the hybridization of the A and B genomes (maybe more than 100,000 years later) (Marcussen et al., 2014), and therefore the diversity of the D-genome is lower (Marcussen et al., 2014; Nielsen et al., 2014). This is most likely why the region located on chromosome 5D, which was significantly associated with Zeleny sedimentation, only contained two genotyped SNPs, whereas associated regions on other chromosomes contained several linked SNPs. The puroindoline genes *Pina-D1* or *Pinb-D1*, which control grain hardness, are located on 5D and have been shown to be associated with several wheat quality traits including Zeleny sedimentation (Bhave and Morris, 2008; Mohler et al., 2012), so the SNPs identified on chromosome 5D in the present study could likely be located in or linked to one of these genes.

Regions associated with Zeleny sedimentation were also found on chromosomes 1B and 1D. Known loci that can affect gluten quality on these two chromosomes are the glutenin loci *Glu-B1* and *Glu-B3, Glu-D1* and *Glu-D3*, the gliadin loci *Gli-B1* and *Gli-D1* (Liu et al., 2014), and the transcription factor *SPA* (*Storage Protein Activator*) (Guillaumie et al., 2004). Several studies have identified QTL for Zeleny or SDS (sodium dodecyl sulfate) sedimentation on chromosome 1A, 1B, and 1D (Deng et al., 2015; Liu et al., 2016; Würschum et al., 2016; Krystkowiak et al., 2017). The QTL most significantly associated with Zeleny sedimentation identified by Krystkowiak et al. (2017) was located on chromosome 5D like in the present study. Sedimentation and other quality traits can also be affected by epistatic interactions between alleles of different loci, but to a smaller extent than by additive genetic effects (Würschum et al., 2016; Krystkowiak et al., 2017). The 1BL.1RS wheat-rye translocation, which has been widely used in European wheat breeding programs (Graybosch, 2001), influences wheat quality negatively, since lines with this translocation lose the *Glu-B3* and *Gli-B1* loci among others. However, none of the 635 lines used in this study contain this translocation (results not shown).

In accordance with the present study, QTL associated with falling number have previously been found on chromosomes 4D and 7B. Mohler et al. (2014) showed that the *b*-allele of the dwarfing gene *Rht-D1* on chromosome 4D increased falling number. In addition, they found a QTL on chromosome 7B, which harbors the α-amylase gene α*-Amy-B2* and a QTL associated with late maturity α-amylase content (Mrva and Mares, 2001). Falling number can be considerably affected by environmental effects, such as the temperature or amount of rain before and during the harvest period. The QTL identified in the present study on chromosome 4D was only significantly associated with falling number, when analyzing only the lines from set2014. This might indicate that this QTL is environment specific, since it was polymorphic in both sets.

The allele frequencies of the SNPs positively associated with Zeleny sedimentation on chromosome 1B, 1D, and 5D were 64 %, 14 %, and 28 %, respectively. Hence, selection of lines based on these three SNPs can significantly improve the Zeleny sedimentation in the breeding material. For TKW, the frequency of the positive allele was 97% for the most significant SNP on 1B, so the allele is almost fixed in the material. Most of the wheat lines had the positive alleles of the SNPs on chromosome 4D and 7B associated with falling number, as the allele frequencies of these two SNPs were 77 and 89%, respectively. Thus, if only marker-assisted selection is used, there is mainly potential for improving Zeleny sedimentation in the studied population. This was also confirmed by the predictions using few associated SNPs, where the difference between using few or all SNPs were smaller for Zeleny sedimentation than for the other traits.

The additive genetic variance explained by the best SNPs were considerably lower when estimated using the Bayesian Power Lasso than when using single marker regression. SNP effects are shrunken to fit with the overall genetic variance, when fitting all SNPs simultaneously in the Bayesian Power Lasso. Conversely, the SNP effects might be overestimated when performing single marker regression because of the Beavis effect (Beavis, 1998; Xu, 2003). The predictive abilities based on the three or ten best SNPs were in most cases highest when selecting the SNPs with single marker regression and then re-estimating the effects of the selected SNPs using a SNP-BLUP model. An explanation for this could be that the standard errors of the SNP effects are taken into account, when selecting based on the significance of the SNP-trait associations. In addition, the effects might be more accurately estimated, when the SNPs are fitted simultaneously rather than separately. For Zeleny sedimentation, the predictions were slightly better, when selecting SNPs using the Bayesian Power Lasso model, indicating that this model might work better for traits controlled by QTL with large effects.

Since the variance explained by single SNPs was quite low for most of the studied traits, genomic selection seems to be a more promising strategy than selection based on few markers. For all traits, the predictive ability increased when using all markers compared to using the three or ten best markers. Other recent studies have reached the same conclusion. Würschum et al. (2016) reported similar predictive abilities as in the present study for protein content and SDS sedimentation when using only significantly associated markers and when using all markers. For test weight, TKW and protein content, using all markers rather than one or five QTL resulted in large improvements in predictive abilities (Norman et al., 2017). Similar results were observed in hybrid wheat, where predictive abilities for seven quality traits generally improved when lowering the significance threshold for markers included in the prediction models (Liu et al., 2016).

One way to implement genomic selection in breeding programs is to select the best lines from a new set (breeding cycle) based on phenotyped lines of previous sets. Only six lines (of a total of 96 different parents) were used as crossing parents for both sets used in the present study. Nevertheless, the principal component analysis and G-matrix showed that the lines of the two sets were genetically related. The average genetic distance between the sets (0.76) was only slightly higher than within sets (0.74). This indicates that the crossing parents used for each set were genetically related. The genetic distance calculated as modified Rogers' distance can range from 0 to 1 (Reif et al., 2005). Here, the maximum distance between two lines was 0.91, and the average distance between lines in the largest groups of full-sibs (33 lines) was 0.51.

The LSO cross-validations resemble how the predictions would perform most realistically, when predicting lines of a new set before phenotypic information is available for those lines. However, the correlations based on the LSO were not as high as for the other types of cross-validations, because of a combination of a smaller training set size, lower genetic relationship between lines of the training and validation sets, and GxE interactions. The k-fold cross-validations showed that lowering the number of lines in the training set had a small negative impact on the correlations. Thus, the main reasons for the lower correlations of the LSO compared with the LOO and LFO were the decrease in genetic relationship between lines and GxE interactions. However, the effect of the genetic relationship may partly be confounded with the GxE interactions. Including data from more years, would most probably improve the results, which is the case in a recent study with spring wheat, where the predictive abilities for several quality traits increased as lines from more years were included in the training set (Battenfield et al., 2016). The cross-validations of the present study indicate that predicting GEBVs of lines from a new breeding cycle based only on lines from a previous cycle might not be the most effective implementation strategy if the predicted traits are strongly affected by GxE interactions. Additional data from lines replicated across locations and/or years would be beneficial in order to account for GxE interactions. Furthermore, the predictive abilities increases if lines from the same breeding cycle, preferably sister lines, are included in the training set. Another way to increase the predictive abilities could be to include pedigree information in the models, although the increase is largest if few markers have been used to estimate the genetic relationship between lines (Cericola et al., 2017).

Bayesian models often perform better than GBLUP models when the genetic relationship between training and validation sets are low, because they are better at capturing LD between markers and QTL (Gao et al., 2013). This improvement is most pronounced for traits that are controlled by QTL with large effects, since Bayesian models allow stronger shrinkage of small effects and weaker shrinkage of large effects compared to GBLUP.

Here, the largest improvement was for the LSO cross-validations for Zeleny sedimentation, when using the Bayesian Power Lasso model instead of the GBLUP model. Thus, the Bayesian Power Lasso model is mainly advantageous to use, if the genetic relationships between lines are low and for traits, where some QTL have large effects.

## Conclusion

SNPs significantly associated with Zeleny sedimentation were identified on chromosome 1B, 1D, and 5D, where genes that are known to affect baking quality of wheat are located. These three SNPs together explain 18.9% of the additive genetic variance according to the single marker regression analysis. The same genetic regions were identified using the Bayesian Power Lasso. However, the explained variance estimated in this model was considerably lower. The prediction of phenotypes based on the best three or best ten SNPs found using the two different methods revealed that the SNPs found using the single marker regression could more accurately predict the phenotypes in most cases. Predictions improved for all traits when using ten SNPs instead of three. The GWAS and predictions using few SNPs indicated that there was a couple of QTL with large effect on Zeleny sedimentation, while the other quality traits seemed to be controlled by many QTL with small effects. Genomic predictions based on all SNPs further improved the predictions and worked quite well for most traits. The predictive abilities were highest for Zeleny sedimentation, while grain protein content was the most difficult trait to predict, especially across breeding cycles. The different types of cross-validations indicated that the genetic relationship between lines and GxE interactions were more important for the predictive abilities and for the bias than the size of the training set. However, the predictions are based on single replication of lines, so additional replications across years and locations would be useful to study the effects of the GxE interactions.Using Bayesian Power Lasso models resulted in similar or slightly higher predictive abilities than when using GBLUP models.

## Author contributions

PK: performed most of the phenotyping, data analysis and wrote the draft for the manuscript; All authors helped designing the study, interpreting results, and reviewing the manuscript. Data was acquired by JA, JO, and PK. Funding was acquired by AJ, JA, JJ, and PK.

### Conflict of interest statement

The study was performed in a collaboration between Aarhus University and the plant breeding company Nordic Seed A/S. Authors PK, AJ, JA, and JO were employed by company Nordic Seed A/S. The other authors declare that the research was conducted in the absence of any commercial or financial relationships that could be construed as a potential conflict of interest.
